# Effects of Maternal Depression and Sensitivity on Infant Emotion Regulation: The Role of Context

**DOI:** 10.3390/children12101323

**Published:** 2025-10-02

**Authors:** Nanmathi Manian, Sandrine Nyivih, Victoria Manzo, Ibilola Adewunmi, Marc H. Bornstein

**Affiliations:** 1 Department of Psychology, University of Maryland–Baltimore County, Baltimore, MD 21250, USA; snyivih1@umbc.edu (S.N.); vmanzo1@umbc.edu (V.M.); ibilola1@umbc.edu (I.A.); 2 Westat Inc., Rockville, MD 20850, USA; 3 Eunice Kennedy Shriver National Institute of Child Health and Human Development, Bethesda, MD 20892, USA; marc.h.bornstein@gmail.com; 4 Institute for Fiscal Studies, London NW1 4DF, UK; 5 United Nations Children’s Fund (UNICEF), New York, NY 10017, USA

**Keywords:** maternal depression, postpartum depression, maternal sensitivity, emotion regulation, fear, still-face

## Abstract

**Highlights:**

**What are the main findings?**

- Maternal sensitivity moderated the association between maternal depression and infant gaze aversion during the still-face episode with the mother and both gaze avert and object-attend during a Fear task as early as 5 months of age.
- The context of the stressor mattered, especially for infants of depressed mothers; they showed reduced use of the self-soothing strategy in the high-arousal fear task, which was a different pattern from their behavior in the still-face paradigm.

**What are the implications of the main findings?**

- Different types of regulatory behaviors might be important and effective for infants in different contexts, including high and low arousal, as well as social and non-social settings.
- Interventions aimed at improving maternal sensitivity—the ability to respond warmly and contingently to her infant’s cues—could be an effective way to improve emotion regulation in infants who are at risk due to maternal depression

**Abstract:**

Introduction/Background: Maternal depression is a significant risk factor for infant emotion regulation (ER), often linked to detrimental mother–infant interactions. Individual effects of maternal depression and maternal sensitivity are known, but their combined influence on infant ER across different emotional contexts remains underexplored. This study investigates concurrent relations among maternal depression, maternal sensitivity, and infant ER in low- and high-arousal contexts in a matched sample of primarily White educated mothers. Methods: We examined 5-month-old infants of clinically depressed and nondepressed mothers. Maternal sensitivity was coded from home observations; infant ER behaviors (e.g., gaze aversion, object-attend, self-soothing) were assessed through observation during modified Still-Face Paradigm (SFP) and fear-eliciting tasks. Results: Clinically depressed mothers exhibited lower maternal sensitivity than nondepressed mothers. Infants of depressed mothers used adaptive ER strategies less—specifically, lower monitoring and gaze aversion in the SFP, and lower gaze aversion and object-attend in the Fear task. Maternal sensitivity moderated the association between maternal depression and infant gaze aversion during the SFP and both gaze avert and object-attend during the Fear task. There was a context-specific regulatory difference for self-soothing; only infants of depressed mothers used self-soothing significantly more during the high-arousal Fear task. Conclusions: These findings underscore the interplay between maternal clinical depression and sensitivity in affecting infant ER. Maternal sensitivity acts as a crucial buffer against the adverse effects of maternal depression on infant ER. The results also indicate that infant emotion regulation varies in different contexts of low and high arousal. Interventions that target maternal sensitivity could significantly improve emotion regulation in infants of depressed mothers.

## 1. Introduction

Maternal depression is a significant risk factor for regulatory disturbances in infants, particularly in the first year of life [1]. Postpartum depression (PPD) is strikingly common—affecting 10–20% of mothers worldwide and about 13% in the United States—making it one of the most prevalent adverse outcomes of pregnancy [2]. The consequences of PPD extend beyond the mother’s well-being—PPD consistently correlates with adverse mother–infant interactions [3,4], which, in turn, link to adverse infant developmental outcomes, including psychophysiological stress responsivity, dysregulation, language deficits, and socioemotional difficulties [1,5,6,7,8,9]. Additionally, early infancy, which roughly corresponds to the first 6 months of life, is a critical period for the development of internal and effortful self-regulation mechanisms, making this period especially vulnerable to the adverse effects of maternal depression and non-optimal parenting [10,11,12]. At around 5 months, infants begin shifting from reliance on caregivers to more intentional self-regulatory behaviors, making this age a key window for studying the early emergence of emotion regulation [13].

Although substantial evidence exists on the socioemotional consequences of being reared by a depressed caregiver, less is known about how maternal depression and maternal sensitivity together contribute to infant emotion regulation. The current study examines concurrent relations among maternal depression, maternal sensitivity, and emotion regulation (ER) in 5-month-old infants in the still-face and fear-eliciting contexts, which are commonly used tasks in assessing individual differences in infant ER. We investigate how maternal depression and maternal sensitivity affect the infant’s ability to regulate emotion, using clinical definitions of depression, naturalistic observations of maternal sensitivity, and assessments of infant ER in a laboratory setting.  

### 1.1. Emotion Regulation in Infancy

Emotion regulation (ER) is the process of modulating and managing internal arousal in response to heightened emotions through developmentally appropriate mechanisms [11,12,14,15]. The ability to regulate emotions is a critical early developmental milestone, foundational for social adjustment in preschool and across the school years [16] and essential for optimal socioemotional, behavioral, and academic outcomes [13,17]. Conversely, difficulties regulating emotions increase risks for later developmental difficulties [18], externalizing and internalizing problems [13], and anxiety, depression, substance use, and eating disorders [19].  

Infant ER is initially dyadic. In the first months, infants rely heavily on caregivers for regulation [20]. By around 5–6 months, infants demonstrate intentional self-regulatory behaviors such as self-soothing, distraction, and communicative bids for adult help [14,21,22]. This achievement reflects a developmental shift from caregiver-dependent regulation toward co-regulation and, eventually, to autonomous self-regulation with caregiver guidance [22,23,24]. Thus, early caregiver–infant interactions play a central role in supporting the development of infants’ regulatory capacities [25]. Patterns of infants’ regulatory behavior assessed during the SFP during the first year, are early developmental precursors of attachment patterns [26,27,28,29].

Typically, ER is assessed in laboratory settings within aversive contexts that require infants to mobilize regulatory resources to manage high arousal. Commonly used paradigms invoke low or high infant arousal. One such paradigm is the fear-eliciting task, which exposes infants to novel or threatening stimuli (e.g., masks or loud noise) to elicit regulatory strategies such as gaze aversion—disengaging attention from distressing stimuli [12,30,31,32,33]. Infants also use other strategies such as orienting toward their mother, disengaging, reengaging with objects, or employing self-soothing behaviors like thumb-sucking or rocking, which have been shown to regulate arousal [34,35,36,37].  

A second widely used context is the Still-Face Paradigm (SFP; [38]), which introduces a brief period of maternal unresponsiveness, inducing moderate negative arousal by violating infants’ expectations of reciprocal social interaction [23,39]. Infants typically respond with the “still-face effect,” characterized by reduced positive affect, increased negative affect, and gaze aversion [40]. The SFP provides a reliable window onto infants’ emerging regulatory capacities and is sensitive to effects of maternal depression [41]. Although studies have not compared the two contexts within the same sample, the length of the still-face episode and the mother’s presence in the SFP support the idea that it may be less arousing than the Fear task. The Fear task, with its sudden and loud noise, the repetitive nature of the stimulus, and the mother’s absence, likely induces a higher level of arousal [12,32].  

Several theoretical explanations for classic still-face effects on infants have been proposed. A review of these explanations is outside the scope of this article, but most emphasize that the SFP represents a dissonance from typical mother–infant interactions [38,42,43]. Even young infants have already developed clear expectations for mutual social interactions and become distressed when these expectations are briefly disrupted [38]. When anticipated stimulation and contingency are disrupted, infants experience distress and must rely on their own limited regulatory capacities, often reflected in heightened negative affect and increased gaze aversion as the infant has only a limited array of regulatory capacities. Other perspectives emphasize the caregiver’s role as external regulator; during the still-face episode of the SFP, when the caregiver becomes unresponsive, infants are compelled to draw on self-regulatory strategies to manage their arousal [44,45,46].  

Given the developmental shift from caregiver-supported to internal ER, and the importance of the independent effects of maternal depression and maternal sensitivity on ER, it is crucial to understand how these factors interact to shape infant ER. Examining how infants of depressed and nondepressed mothers employ different ER strategies, how maternal sensitivity contributes to the utilization and adaptability of these strategies, and how ER strategies vary across low versus high stress-inducing contexts can deepen our understanding of later ER development [47]. Understanding the relations between these factors has clinical significance, as poor regulation of negative emotions or maternal unresponsiveness in infancy increases risks for later emotional and behavioral problems in children [13,19]. A better understanding of these interactive processes can also support future interventions for optimal development of infant ER outcomes by targeting both caregiver sensitivity and infant self-regulatory capacities [25].

In addition, although infant ER has been studied separately in the SFP and fear-eliciting contexts, little research has examined these paradigms together or how maternal depression and sensitivity influence ER across different arousal contexts. Addressing this gap can deepen our understanding of the expression of ER in infancy and how the environment influences that expression.

### 1.2. Maternal Depression and Infant ER

PPD disrupts early mother–infant interactions, which can hinder optimal emotion regulation development [29,48]. Infants of depressed mothers—sometimes as early as 3 to 6 months—display ER deficits such as limited attention regulation and self-soothing [32,40,47,49,50]. ER strategies are learned within caregiver–infant interactions, which tend to be less sensitive and contingent when mothers are depressed [51], and infants may also model their parents’ maladaptive strategies [52].  

Less is known about how maternal depression shapes infant ER in fear-eliciting contexts, but existing evidence points to infants’ greater reliance on “immature” strategies, such as thumb-sucking [12,32]. Some work has examined ER in the SFP, though the findings are inconsistent. Theoretically, the SFP might be less distressing for infants of depressed mothers because the unresponsive and flat affect displayed then mirrors their daily interactions [53], potentially dampening distress and reducing the need to self-regulate. Alternatively, chronic exposure to non-optimal interactions with a depressed mother may impair the development of more sophisticated ER strategies, limiting infants’ use of gaze aversion or self-soothing. Empirical results on infants’ regulatory responses during the SFP are inconsistent—some studies report similar SFP effects across the two groups [29], whereas others find differences between infants of depressed and nondepressed mothers in negative affect, gaze aversion, or even increased positive affect as a re-engagement strategy [54,55,56,57,58]. In a previous study, we observed no group differences between infants of depressed and nondepressed mothers in affect or the use of ER during SFP, apart from gaze aversion, suggesting that both groups experienced it as distressing, but that infants of depressed mothers may be less able to use attentional regulatory strategies *effectively* (see [40]).

### 1.3. Maternal Sensitivity and Infant ER

A mechanism underlying the association between maternal depression and negative child outcomes are the disruptions in parenting and the quality of mother–child interaction [3,32,59,60,61]. Maternal sensitivity, broadly defined as “timely, contingent, and appropriate responding to infants’ cues” [3] (p. 586), is critical for infant ER. Infants primarily interact with their mothers or other primary caregivers, who act as co-regulators of infant distress, physiology, and behavior [3]. Positive interactions, where caregivers initiate and respond to infant interactions with timely, accurate, and warm verbal, tactile, and visual stimulation, signal the mother’s availability, establish an interaction schema, and serve as a platform on which infants learn self-regulation strategies [62].  

Although maternal sensitivity has been studied in relation to infants’ emotion regulation (ER), findings remain mixed across contexts. In fear-eliciting tasks, for instance, Feldman et al. [32] found no effects in infants, whereas Glöggler and Pauli-Pott [36] reported that greater maternal sensitivity was associated with more self-soothing in 30-month-olds. Research using the SFP also points to inconsistencies. Some studies have shown no relation between maternal sensitivity and infant responses at 3 months [39], whereas others suggest that infants of sensitive mothers are more likely to engage in regulatory behaviors, display more positive affect, and show less avoidance and negative affect during the still-face and reunion episodes [63]. In another study, maternal sensitivity measured during 10 min free play at home correlated with infant vagal regulation across SFP at 6 months. Infants of sensitive mothers were more aroused and distressed by their mothers’ disengagement presumably reflecting the infants’ historical schema of interactions [23]. These findings suggest that maternal sensitivity and maternal depression may exert separate influences on the development of the infant’s ER during the first months of life.

Given maternal sensitivity’s link to infant affect in the SFP, we can expect maternal sensitivity to moderate the relation between depression and infant ER. Indeed, some studies support this moderation, showing maternal sensitivity can buffer the effects of mothers’ mood disorder on infants’ physiological self-regulation at 4 months [64,65] and moderate the association between mothers’ prenatal internalizing disorder and infant attentional regulatory capacity at 6 months postnatal [12]. Although these studies did not directly examine maternal depression and infant ER behaviors, collectively they suggest maternal sensitivity acts as a crucial moderator, buffering the potential negative impact of maternal depression on early infant ER.  

### 1.4. Context in Infant ER

An important factor in understanding infant ER is the context in which distress arises. Studies have shown that infant ER varies by emotional context (e.g., [31,66]), yet the mechanisms underlying these differences remain unclear. While infants often seek comfort from their mothers during distress [67], such efforts may be ineffective when the mother is unresponsive, as may occur in the context of maternal depression [68,69]. SFP and fear-eliciting paradigms, though both widely used, differ markedly in the types of stress they induce. Fear tasks typically involve high-arousal, non-social stimuli that elicit greater reliance on self-regulation, whereas the SFP presents a lower-arousal but longer-lasting stressor in which the caregiver’s unresponsiveness disrupts infants’ reliance on social soothing and prompts the use of alternative ER strategies. Maternal sensitivity may therefore play a greater role in contexts where the mother is present in infant’s visual field (SFP) versus absent (Fear task). In addition, the effectiveness of attention-based strategies may depend on the level of arousal [34,35,50]. Based on prior literature, we expected the fear-eliciting task to elicit higher arousal and the SFP to reflect moderate arousal that is more closely tied to caregiving history, with differences in infant behaviors reflecting both ER skills and prior caregiving experiences. That is, we expected the fear-eliciting task to reveal stronger effects of maternal depression on infants’ regulatory strategies, since its inherently high-arousal context is likely to elicit distress independent of prior caregiving experiences.  

### 1.5. Current Study

Our study examines relations among maternal depression, maternal sensitivity, and infant ER at 5 months of age across two different regulatory contexts. We examined infant behaviors during the SFP (a moderate-arousal, caregiver-related stressor) and a fear-eliciting task (a high-arousal, novelty-based stressor). In both contexts, we assessed core infant ER behaviors: attention regulation strategies such as gaze aversion, looking at mother, attending to objects, and self-soothing. Maternal sensitivity, based on extended naturalistic observations in the home, captured multiple dimensions of interaction, including social play, positive affect, and contingent vocalization.

We also coded infant protest in the SFP and infant fear in the Fear task, which involves different negative facial expressions or vocalizations. Whereas infant affective behaviors are not typically considered a primary regulatory strategy, we included them to ensure that our manipulation of infant affect during the SFP and Fear task were accurate and valid, and that each context successfully elicited negative arousal. The primary dependent variables for infant ER were infant monitor, gaze aversion, object attend, and self-soothing in SFP and gaze aversion, object attend, and self-soothing in Fear task.

The aims of the study were to (1) examine the main effect of maternal depression on infant ER behaviors across the SFP and fear-eliciting contexts, (2) examine associations between maternal sensitivity and infant ER behaviors across SFP and fear-eliciting contexts, (3) assess whether maternal sensitivity moderates the relation between maternal depression and infant ER, and (4) investigate how contextual factors influence relations among maternal depression, maternal sensitivity, and infant ER.

Based on the extant literature, for Aim 1 and Aim2, we hypothesized that infants of nondepressed mothers—and infants exposed to more sensitive caregiving—would show better regulatory behaviors across the two tasks, as evidenced by more gaze aversion, object attend, and self-soothing. For Aim 3, we hypothesized that maternal sensitivity would buffer the negative effects of maternal depression on infant ER (i.e., a moderation effect). However, we were less certain about how these effects would vary by context (Aim 4). In general, we expected stronger interactive influence of maternal depression on infant ER strategies in the fear-eliciting task, as it is a high-arousal condition likely to be perceived as negative regardless of the infant’s caregiving history. In contrast, predictions of these associations for the SFP task were more nuanced. Although infants of depressed mothers may show reduced regulatory behaviors (e.g., less gaze aversion or object engagement), it is also possible that they find the still-face episode of the SFP less distressing—particularly if they are accustomed to less sensitive interactions and therefore do not expect or seek maternal engagement.

## 2. Materials and Methods

### 2.1. Participants

Mothers (≥20 years) who could read and speak English were recruited from the Washington DC metropolitan area between 2002 and 2009. We used multiple community-based strategies for recruitment, including mass mailings, outreach through women’s organizations, and newspaper advertisements. These approaches were designed to reach a broad cross-section of the local population and to ensure adequate representation of mothers who met eligibility criteria. Mothers interested in participating in the study completed the Beck Depression Inventory-II (BDI-II; [70]), a validated 21-item self-report questionnaire. Each item reflects a particular symptom of depression and aligns to the diagnostic criteria listed in the Diagnostic and Statistical Manual of Mental Disorders (4th ed.; DSM-IV; [71]). From the 667 mothers who returned the BDI-II, 446 mothers with low (1–7) and high scores (>12) (see [72] for details) were contacted to participate between 3.5 and 5 months postpartum to complete the Structured Clinical Interview for DSM-IV Axis I Disorders (SCID-I; [73]). The SCID-I was then conducted by certified mental health professionals. Mothers diagnosed as having had Major Depressive Disorder (MDD) or Minor Depressive Disorder (mDD) within the lifetime of the infant were placed into the depressed group, and those without any such diagnosis were placed into the nondepressed group. All infants were singletons, born within 3 weeks of their due date, and were healthy at birth and at the time of the observation.  

All procedures with human participants were reviewed and approved by the Institutional Review Board (IRB) at the National Institutes of Health (NIH) Clinical Center, *Eunice Kennedy Shriver* National Institute of Child Health and Human Development under Protocol 02-CH-0278 (NIH Clinical Trials Identifier: NCT00044174).

For the current study, we used matched groups of 60 clinically depressed and 60 nondepressed mothers (see methods in [5]). Matching was conducted using 2 × 2 tables with infant gender and parity for available depressed and nondepressed groups separately. Within each of the four cells, maternal average age, education (less than standard college, college, graduate or higher), race/ethnicity (European American, African American, other), and SES (average family income) were classified. Nondepressed mothers who matched for these characteristics within each cell were selected for the corresponding cell for the depressed sample.

Mothers’ ages ranged from 21 to 43 years (*M* = 31.57, *SD* = 4.52); their infants ranged from 20 to 23 weeks (*M* = 21.74, *SD* = 0.80). The majority (90.8%) of the mothers were married. Furthermore, 69.7% of the mothers identified as White, 13.0% as African American/Black, 9.2% as Hispanic, 4.9% as Asian, and 3.2% as mixed or another race/ethnicity. For education, 20.2% of the mothers had partial college or less, 42.0% had completed college, and 37.8% had completed graduate programs. Of the mothers in the depressed group, 9 (15%) had co-morbid Axis I disorders, primarily Generalized Anxiety Disorder (GAD); and 15 (25%) were currently on medication.

None of the infants had known genetic disorders or birth complications. Most were firstborn (61.6%), and the sample was balanced by sex, with boys comprising 51% of the depressed group and 58% of the nondepressed group.

### 2.2. Procedures

The procedures involved home and laboratory visits to assess maternal sensitivity and infant ER, respectively. The two visits were scheduled when the infants were healthy; home visits were conducted prior to laboratory visits. We followed the informed consent procedures and obtained written consent from the mothers for their participation and their infant’s participation in the home and lab visits.  

Maternal Sensitivity (Home Visit): When infants were 5 months of age, each mother–infant dyad was visited in their home by a single observer, and an hour-long video record of the dyad’s naturalistic interaction was made. After the mother received instructions, time was provided for her and the infant to acclimate to the presence of the videographer and camera [71,72], allowing them to settle in and behave as naturally as possible in their familiar home environment. The videographer resisted talking to the mother or making eye contact with, interacting with, or otherwise reacting to the infant during the filming. Dyads were video recorded at a time when the infant was awake and alert. The mother was instructed that the videographer was interested in her and her infant’s going about their usual activities at a time when the mother was at home and solely responsible for the baby. The mother was asked to disregard the videographer insofar as possible. These naturalistic observations captured a range of routine mother–infant daily activities, such as bathing, feeding, diapering, soothing, joint play, and so forth.  

Infant emotion regulation (SFP): We used a modified SFP [74] in a laboratory setting when the infant was 5 months old. The infant was seated at eye level across from the mother at eye level, with no toys present. Three cameras recorded the interaction: wo capturing different angles of the infant and one focused on the mother These recordings were combined into split screen videos with digital time codes. The SFP consisted of three episodes. The first was a 3 min naturalistic interaction (Baseline), in which mothers were instructed to “play with your baby naturally, like you would at home.” The second was a 2 min modified still-face episode, during which mothers were asked to interact as they might on days when they felt tired and unable to engaged effectively with their infant—maintaining gaze, speaking in a flat monotone, keeping their faces expressionless, and minimizing movement and touch (see [74]). Prior to the task, mothers observed a standardized demonstration of this behavior. Thus, in this study, the still-face episode deviated from the traditional “no talk, no touch, but maintain eye contact” protocol by adopting a more naturalistic approach intended to approximate how depressed mothers might interact at home with their infants. The final episode was a 2 min Recovery episode where the mother returned to natural play. The current study is based on infant behaviors observed only during the modified still-face episode.  

Infant emotion regulation (Object Fear Task): Drawing from previous studies that assessed infant ER during a fear-eliciting task, infants completed the object Fear task from the Laboratory Temperament Assessment Battery (Lab-TAB; [75]) at the 5-month lab visit. Lab-TAB is an assessment designed to measure infants’ reactions to stimuli that elicit emotional or behavioral reactions across broad dimensions of infant temperament (see [76]). Based on piloting with different stimuli for 5-month-old infants, we selected the Loud Noise episode wherein a sudden loud noise emanating from a hair dryer (selected because of high ecological validity) are presented to the infant sitting in a highchair in front of an enclosure with a curtain, that played for 3 trials for 10 s (includes 5 s of loud noise and 5 s without noise). Mothers sat at the back of the highchair and were instructed not to comment or react to the noise or to their infants’ responses. Mothers could stop the procedure at any time. Although no studies have employed the exact Fear task methodology we used from the Lab-TAB manual, which is widely applied in temperament research, studies have used loud sounds to elicit fear [77,78,79].  

### 2.3. Data Coding and Scoring

All maternal and infant behaviors were coded by raters blind to participant diagnosis and study hypotheses. There were no missing values in the dataset.  

#### 2.3.1. Maternal Sensitivity

The digitized video records from the home visit were coded using mutually exclusive and exhaustive coding schemes and real-time observation coding procedures [80,81]. Coders made separate passes to record codes for maternal behaviors (maternal vocalization, maternal play/positive evaluation). For all cases, the total length of the video record that could be coded was more than 48 min. If codable time totaled less than 50 min, data from that case were prorated. Coders were first trained to reliability on consensus coding, and between 18% and 25% of the sample was coded independently to obtain reliability (*kappa*; [82,83]).  

Mother Vocalization: Mother vocalization to infant included three domains. Infant register (ĸ = 0.69) with speech/speech-like sounds that are directed to the infant and are characterized by high-pitch or fluctuation in pitch; Conversational tones (ĸ = 0.71) and words/speech-like sounds directed to the infant and characterized by normal intonations and singing; and Imitation (ĸ = 0.65) of infant’s nondistress or distress vocalization that is contingent and similar in intonation pattern.  

Mother Positivity: Mother positivity used two domains, the social play (ĸ = 0.73) was the mother physically or verbally engaging in activities intended to amuse the infant or to elicit smiles, positive vocalizations, laughter, or motoric excitement, and express affection (ĸ = 0.67) was the mother physically or verbally expressing affection or positive affect to the infant.  

We converted the data to proportions of total interaction time and then aggregated them into a composite score for maternal sensitivity, as the mother vocalization and mother positivity were coded as mutually exclusive codes of duration. To account for the fact that the means and variances of proportions are not independent and the behaviors were not normally distributed, all proportions were logit transformed for analysis [84].

#### 2.3.2. SFP  

The present study focused on infant behaviors observed during the still-face episode of the SFP. Infant affective states were coded as coherent configurations of facial expressions and behaviors rather than as discrete behaviors (see [46]). The coding system drew on the Infant Regulatory Scoring System (IRSS; [85]) and the AFFEX system [86]. Infant states were categorized into mutually exclusive and exhaustive codes: protest (negative facial expression, vocalization), monitor (gaze at mother’s face with neutral expression or vocalization), gaze aversion (active gaze away from mother’s face or objects with neutral or slightly negative facial expression), and object attend (focused attention on objects in the SFP setting). Video recordings were coded in 1 s time intervals. Self-soothing was coded separately as active mouthing or repetitive manipulation of a body part or object lasting more than 1 s.  

Coders were trained to reliability using pilot video recordings of mother–infant interactions. Reliability was defined as both coders independently coding the same behavior within 1 s, and assessed with kappa (κ) for 10% of depressed and 10% of nondepressed dyads. For infant states, the κ was 0.79 (depressed group) and 0.88 (nondepressed group). For self-soothing, κ was 0.86 (depressed group) and 0.84 (nondepressed group).

For the SFP, the dependent variables were the durations of infant monitoring, gaze aversion, object attending, and self-soothing. These behaviors were coded as mutually exclusive durations and converted to proportions of total interaction time. Because the distributions were non-normal and proportions have interdependent means and variances, the variables were logit-transformed for analysis [84]. Thus, the final dependent variables were logit-transformed proportions of infant ER behaviors.

To conduct manipulation checks, we coded mothers’ behavior across the three SPF episodes to assess whether depressed and nondepressed mothers complied with instructions and maintained a still face. Maternal facial, vocal, and tactile expressions of positive (κ = 0.82) and neutral (κ = 0.78) behaviors were coded by coders independent from those coding infant states. The success of the still-face manipulation was evaluated by examining maternal affective behavior during the SFP task. We conducted a 2 (depressed and nondepressed group) X 3 (SFP episodes) repeated-measures GLM showed a significant change in maternal neutral behaviors, *F*(2,117) = 27.23, *p* < 0.01, and in maternal positive behaviors, *F*(2,117) = 26.23, *p* < 0.01, across episodes. There were no significant differences in neutral expressions, *F*(1,118) = 1.74, ns, or positive expressions, *F*(1,118) = 0.23, ns, between the depressed and nondepressed mothers during the SFP. These results indicate that the manipulation was effective for both groups, as depressed and nondepressed mothers displayed comparable behaviors during the SFP.

#### 2.3.3. Fear

Coding of infant emotion regulation was adapted from Stifter and Braungart [87] and to align with SFP coding. Trained research assistants coded intensity of facial fear (0 = no facial regions show codable fear movement) to 4 (all three regions of the face show fear accompanied by crying), distress vocalizations (0—no distress to 3—definite non-muted crying), bodily fear (0-no sign of bodily fear, to 1—decreased activity or tensing/freezing/trembling). All three dimensions were rated every 5 s. The emotion regulation behaviors were coded as present (1) or absent (0) in 5 s epochs across each of the 3 trials, for a total of 6 epochs. ER behaviors were aggregated across the trials and reduced into three behaviors: gaze aversion, object attend, self-soothing. Across assessments, intensity of fear facial expression, distress vocalization, and bodily fear ratings were summed to form a fear composite. Average coding reliability for the Fear episode was average ĸ = 0.76.

In order to do a manipulation check of whether the infants found the trial to be fearful, we derived a composite of fear by summing across infant facial fear, bodily fear, and vocalization. The infant codes of fear, gaze avert, object attend, self-soothe were retained as the raw codes of the aggregated frequency of the behavior. We did not find extreme values in the dataset or non-normal distributions of the study variables using Shapiro–Wilk’s tests [88].

Coders were first trained to reliability using pilot recordings of infants, after which a subset of 10% of depressed and 10% of nondepressed dyads (12 video records in total) was selected for reliability testing. Reliability was defined as both coders independently coding the same behavior as a ‘0′ (absent) or ‘1′ (present) within an epoch, with average reliability (κ) values of 0.74 for the depressed group and 0.79 for the nondepressed group.

The dependent variables for infant ER behaviors for the Fear task are the aggregated frequencies of gaze avert, object attend, and self-soothing.

## 3. Results

Preliminary analysis showed that the matched groups of infants of clinically depressed mothers (*n* = 60) and infants of nondepressed mothers (*n* = 60) did not differ on any infant or maternal sociodemographic variables (Table 1), except for birth order, which was therefore examined for possible main or interactive effects on maternal or infant variables. Birth order was associated with maternal depression, χ^2^(1) = 4.17, *p* = *0*.041; 68.3% of depressed mothers had firstborns participate in this study as compared to 50% of the nondepressed mothers. However, birth order did not correlate with infant ER or maternal sensitivity. Analyses were therefore conducted with group as the single factor to maximize power to detect effects for depressed versus nondepressed group status as the focal independent variable.  

In addition, a univariate GLM indicated a significant effect of maternal depression on maternal sensitivity, *F*(1,118) = 4.21, *p* = 0.042, η^2^ = 0.034. Depressed mothers at 5 months displayed lower sensitivity (*M* = −1.62, *SD* = 0.055) than nondepressed mothers (*M* = −1.46, *SD* = 0.055).

### 3.1. Aim 1: Main Effects of Maternal Depression on Infant ER

We conducted univariate ANOVAs with maternal depression as in the independent variable and infant regulatory behaviors during SFP and the Fear task as the dependent variables. The results are presented in Table 2. There were main effects of depression on infant behaviors during SFP. Maternal depression had a significant effect on proportion of time infants engaged in monitor (*F*(1,118) = 7.01, *p* = 0.009, η^2^ = 0.06) and gaze aversion (*F*(1,118) = 8.54, *p* = 0.004, η^2^ = 0.07). Infants of depressed mothers had lower proportions of monitor (*M* = −0.96, *SD* = *0*.08) and lower gaze aversion (*M* = −0.95, *SD* = 0.07) as compared to infants of nondepressed mothers (*M* = −0.67, *SD* = 0.08, and *M* = −0.68, *SD* = 0.06, respectively). There was no difference in the proportion of time infants spent in object attend or self-soothing; infants of both depressed and nondepressed mothers showed similar levels of protest, object attend, and self-soothing behaviors.  

We conducted similar analyses for infant fear and for the infant regulatory behaviors during the Fear episode. There were main effects of depression on infant behaviors. Maternal depression had a significant main effect on infant fear (*F*(1,118) = 4.29, *p* = 0.040, η^2^ = 0.04), gaze aversion (*F*(1,118) = 5.76, *p* = 0.018, η^2^ = 0.05), and object attend ((*F*(1,118) = 6.54, *p* = 0.012, η^2^ = 0.05), such that infants of depressed mothers were more fearful (*M* = 0.96, *SD* = 0.09) but showed lower frequencies of gaze aversion (*M* = 0.52, *SD* = 0.03) and object attend (*M* = 0.78, *SD* = 0.08) as compared to infants of nondepressed mothers. There was no significant difference in the infant self-soothing behaviors.

### 3.2. Aim 2: Associations Between Maternal Sensitivity Infant ER

We examined bivariate correlations between maternal sensitivity and infant behaviors in both the SFP and Fear task (see Table 3). Across the two groups of infants of depressed and nondepressed mothers, maternal sensitivity was positively related to infant monitor (r (118) = 0.18, *p* = 0.044), gaze aversion during SFP (r (118) = 0.28, *p* = 0.022); gaze aversion (r (118) = 0.45, *p* < 0.001), and object attend (r (118) = 0.30, *p* < 0.001) during the Fear task. No significant correlations emerged among the same infant regulatory behaviors across situations.

### 3.3. Aim 3: Maternal Sensitivity as a Moderator of the Relation Between Maternal Depression and Infant ER

We conducted stepwise multiple regression analysis to examine the interactive effects of maternal depression and sensitivity in predicting infant regulatory behaviors. In step 1 of the regression equation, we entered maternal depression and sensitivity, and in step 2, we added the interaction term between maternal depression and sensitivity (see Table 4).

**Figure 1:**
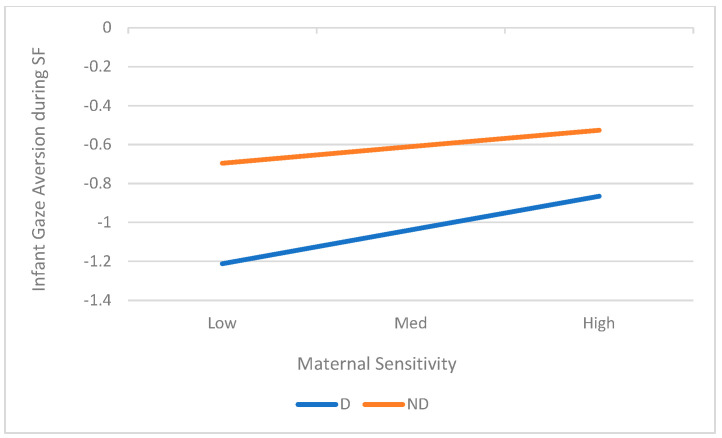
Interaction between Mother Depression and Sensitivity on Infant Gaze Aversion During SFP Task.

As can be seen from Table 4, there were significant Maternal Depression X Maternal Sensitivity interactions for infant gaze aversion (*B* = 0.63, *p* = 0.019, *R*^2^ change = 0.13) in the SFP context. To explore the interaction, we conducted simple ANOVAs in the two groups of depressed and nondepressed. Although both groups showed greater gaze aversion with increased sensitivity, the slope was higher for infants of depressed mothers (*B* = 0.39, *p* = 0.026, *R*^2^ = 0.29). The interaction is shown in Figure 1.

**Figure 2:**
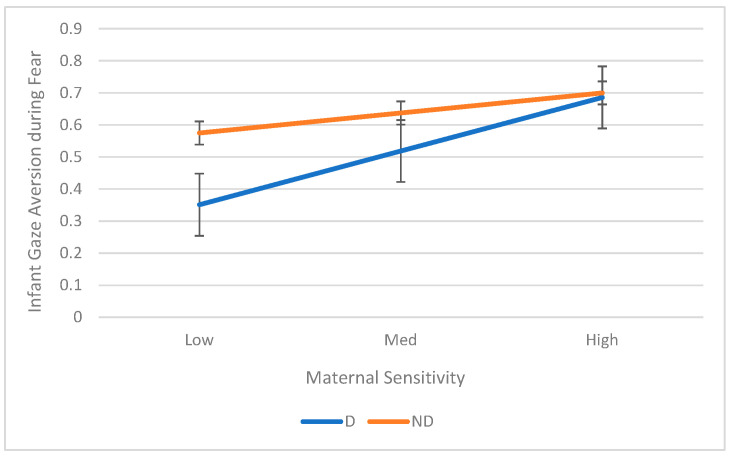
Interaction between Mother Depression and Sensitivity on Infant Gaze Aversion During Fear Task.

The interaction between maternal depression and sensitivity was significant for infant gaze aversion (*B* = 0.27, *p* = 0.016, *R*^2^ change = 0.26) during the Fear task. To explore the interaction, we conducted simple ANOVAs in the two groups of depressed and nondepressed. Infants of depressed mothers showed increased gaze aversion with increased maternal sensitivity (*B* = 0.42, *p* < 0.001, *R*^2^ = 0.69). The interaction is shown in Figure 2.

**Figure 3:**
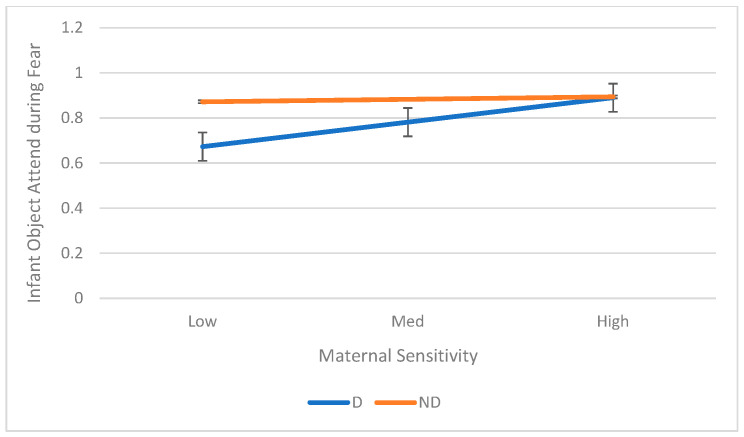
Interaction between Mother Depression and Sensitivity on Infant Object Attend during Fear Task.

There were significant Maternal Depression X Maternal Sensitivity interactions for infant object attend (*B* = 0.25, *p* = 0.010, *R*^2^ change = 0.17) during Fear task. Simple ANOVAs showed that infants of depressed mothers showed increased object attend with increased maternal sensitivity (*B* = 0.28, *p* < 0.001, *R*^2^ = 0.43). The interaction is shown in Figure 3.

### 3.4. Aim 4: Role of Context on Infant ER

We used a generalized linear model (GLM) mixed-model approach with “context” as the repeated measure to examine contextual effects on infant ER strategies (Aim 4). Because measurement units differed between the SFP (logit transformed proportion of duration) and Fear (aggregated frequencies) codes, we converted the scores to z-scores to reflect each infant’s ER strategies relative to the sample mean. We used the infant ER z-scores in the two contexts as the repeated-measures variable and entered the context and its interactive effects with maternal depression and sensitivity entered in sequential steps (see Table 5).

There was a significant Context X Depression interaction on infant self-soothe (*F*(1,118) = 3.95, *p* = 0.049, η^2^ = 0.03), such that there was a differential effect of maternal depression on infant self-soothe in the Fear task (*B* = 0.46, *p* = 0.012) but not in the SFP (*B* = −0.04, *p* = 0.828). That is, the use of the self-soothing strategy was similar for both groups of infants in SFP but significantly higher in the Fear task for infants of depressed mothers.

## 4. Discussion

This study examined how maternal depression and maternal sensitivity influence infant emotion regulation (ER) at five months of age across two contrasting arousal contexts: the SFP and a fear-eliciting task. Our findings extend prior research by showing that maternal depression, sensitive mother–infant interactions and the nature of the stressor influence infants’ regulatory behaviors in a sociodemographically matched sample, and that maternal sensitivity can buffer some of the negative effects of maternal depression—particularly for attentional strategies in high-arousal settings.

Consistent with our hypotheses and prior research, maternal depression was associated with lower maternal sensitivity and poorer infant ER outcomes [3,89]. Research has shown that core symptoms of clinical depression—such as low mood, anhedonia, fatigue, and irritability—may diminish expressions of maternal warmth, responsiveness, and engagement, resulting in less synchronous and at times more withdrawn or intrusive mother–infant interactions [3]. These interactional difficulties can limit opportunities for infants to receive consistent modeling and guidance in ER, thereby constraining the development of active regulatory skills [49,62]. In our study, infants of clinically depressed mothers showed reduced reliance on adaptive attentional strategies: specifically, less monitoring and gaze aversion during the SFP, and reduced gaze aversion and object attending during the Fear task. Notably, these differences emerged even though infants of depressed and nondepressed mothers displayed comparable distress in the SFP, and despite infants of depressed mothers finding the Fear task more frightening overall. That is, the group differences cannot be explained by variations in distress levels; rather, they suggest that infants of depressed mothers have less developed regulatory capacities. In high-arousal contexts, these infants engaged less in strategies such as gaze aversion and object attending—behaviors that could otherwise help them manage negative arousal.

Maternal sensitivity was positively associated with several adaptive infant ER behaviors—monitoring and gaze aversion during the SFP and gaze aversion and object-attend during the Fear task. Our sensitivity construct, based on extended naturalistic observations in the home, captured multiple dimensions (e.g., social play, positive affect, contingent vocalization), supporting its ecological validity. In the context of SFP, which is a social stressor, an infant might monitor their mother’s face to try and re-engage her, or they might avert their gaze to manage the distress of the interaction. For infants with sensitive mothers, this relational history may have instilled a sense of confidence in their social environment, making them more likely to use these socially oriented strategies to cope with the disruption. In the fear task, where the object is the stressor, the positive relationship between maternal sensitivity and gaze aversion and object-attend suggests that sensitive caregiving helps infants develop the capacity for self-regulation and environmental exploration, which they can then apply to non-social, high-arousal situations. Research on maternal sensitivity during the first year, particularly in contexts of child distress, shows that high sensitivity is linked to better child ER, both concurrently and over time [90,91]. Consistent with this, our findings suggest that differences in child ER across contexts are explained by context-dependent variations in maternal sensitivity. The fact that maternal sensitivity is positively associated with different behaviors in each task suggests that sensitive caregiving supports a flexible, context-appropriate repertoire of emotion regulation strategies in infants.

The most compelling findings emerged from the interaction analyses (Aim 3). In the SFP, a significant maternal depression X maternal sensitivity interaction for infant gaze aversion revealed that, whereas increased sensitivity was associated with greater gaze aversion in both groups, this relation was more pronounced for infants of clinically depressed mothers. This finding suggests that, for infants with depressed mothers, higher maternal sensitivity might be particularly crucial in facilitating disengagement from the distressing circumstances, such as the SFP. Even more strikingly, in the fear-eliciting task, significant interactions were observed for both infant gaze aversion and object-attend. For infants of depressed mothers, increased maternal sensitivity was associated with a notable increase in both gaze aversion and object-attend, providing strong evidence that maternal sensitivity buffers the negative impact of maternal depression on these attentional regulatory strategies in a high-arousal situation. These findings regarding the interactive effects of maternal sensitivity echo work showing that maternal sensitivity can buffer the effects of maternal mood disorders on infant physiological and attentional regulation [12,64].

The present study demonstrated similar buffering effects of maternal sensitivity on specific behavioral regulatory strategies. Despite the positive correlation between maternal depression and sensitivity, our study suggests that, for depressed mothers who have a history of interacting with their infant in a sensitive way (e.g., vocalizing and playing with the child in a positive way), this established dyadic history provides a crucial foundation. This relational history, built through sustained, sensitive interactions as measured in our study, appears to enable infants to utilize adaptive regulatory strategies when faced with an emotional challenge. This finding indicates that the protective effects of sensitivity are not merely a reaction to the moment but are a culmination of a history that builds the infant’s capacity for self-regulation [65,92]. When an infant’s intrinsic capacities are limited, they may require greater external regulation from a sensitive caregiver to learn ER strategies.

Our findings align with prior research from Fuertes and colleagues (e.g., [93]), who have demonstrated associations between maternal sensitivity and infant ER using the SFP both full-term and preterm samples. Notably, the pattern we observed resembles findings from studies of preterm infants, whose mothers are often experiencing depression, suggesting that maternal sensitivity may play a similar protective role across different forms of early adversity.

Finally, we found a significant interaction between context and maternal depression for infant self-soothing. The effect was specific to the Fear task—infants of clinically depressed mothers used self-soothing significantly more (relative to the group mean) compared to infants of nondepressed mothers. Self-soothing refers to internally directed behaviors that help regulate tension while supporting continued engagement with the external environment [94]. Although a foundational coping mechanism, self-soothing is considered to be a “primitive” form of emotion regulation by some researchers because it appears earlier in development than other strategies like self-distraction and is less associated with active environmental exploration [95]. High-arousal, non-social stressors may place greater demands on self-regulation when the caregiver is unavailable to co-regulate. Such behaviors tend to decline or lose effectiveness in fear or frustration contexts [87,96,97]. For example, infants of depressed mothers rely more on self-soothing during a fear-eliciting task that was less effective in reducing distress [32], and toddlers (ages 24 and 30 months) used self-oriented strategies (e.g., self-soothing) for fear but other-oriented strategies (e.g., trying to engage the mother) for anger [37]. Our findings confirm this picture in 5-month-old infants: whereas group differences did not emerge in the individual contexts, only infants of depressed mothers showed more self-soothing in the Fear task as compared to the SFP, suggesting that elevated arousal may impair infants’ use of more sophisticated attentional strategies, causing them to resort to self-soothing. While infants use this strategy, it does not seem to be effective, considering they had higher fear scores as compared to infants of nondepressed mothers, consistent with our previous research [40].

The lack of correlation among the same regulatory behaviors across the two contexts reinforces the idea that ER strategies are context-specific rather than fixed traits in accord with the specificity principle [98,99]. SFP and fear-eliciting tasks differ in several ways: the SFP involves the caregiver as the stressor, whereas the Fear task is non-social and typically elicits higher physiological arousal in quick successive episodes [47]. Drawing on Ponzetti et al.’s work [37], it is also possible that the SFP elicited a mix of emotions—including frustration or anger toward the unresponsive mother—which may promote other-directed strategies like monitoring. Indeed, monitoring was higher among infants of nondepressed mothers in the SFP. Because we did not code monitoring in the Fear task (due to the mother’s absence from the infant’s visual field), we cannot yet determine whether infants as young as five months differentially deploy self- versus other-directed regulation in fear versus anger contexts.

## 5. Strengths, Limitations, and Future Directions

This study has several notable strengths. First, we compared depressed and nondepressed mothers matched on sociodemographic characteristics, reducing the need to statistically control for these factors known to influence both maternal depression and mother–infant interactions. Second, maternal depression was identified using objective clinical diagnostic criteria rather than self-reported symptoms. Third, maternal sensitivity was assessed in the home over nearly an hour of naturalistic observation, enhancing ecological validity by capturing interactions in familiar, everyday settings [100]. Fourth, our measure of infant ER was comprehensive and based on detailed observational coding across two different tasks rather than maternal report, offering a more objective account and robust assessment of infant behavior. In sum, our use of clinically diagnosed maternal depression and naturalistic observations of infant ER in two distinct contexts strengthens the ecological validity of our findings.

Potential confounds should also be acknowledged, as maternal comorbidities such as anxiety, paternal/partner involvement in caregiving, and cultural differences in parenting practices may have influenced both maternal sensitivity and infant ER. In addition, research also indicates that children from low-income households may be more vulnerable to difficulties in self-regulation compared to their peers from higher socioeconomic backgrounds [101,102].

Despite these strengths, several limitations should be acknowledged. First, the cross-sectional design at five months limits our ability to establish causality or examine developmental changes in emotion regulation over time. Second, although maternal depression was diagnosed using clinical criteria, the “lifetime of the child” definition may include mothers who were not currently depressed at the time of observation, potentially diluting acute effects on infant regulation. As such, the associations observed here may represent conservative estimates, with true effects possibly being even stronger. Furthermore, our modified SFP, designed to be ecologically valid for depressed mothers, deviates from the traditional protocol, which might affect direct comparisons with some existing literature. The sample, though carefully matched on sociodemographics, infant gender, and parity, consisted primarily of White, educated mothers, and the findings may not be fully generalizable to other populations.

Future research should build upon these findings by employing longitudinal designs to track the developmental pathways from maternal depression and sensitivity to infant ER across time. Exploring a wider range of ER strategies, including physiological measures, would also provide a more comprehensive understanding of the underlying regulatory processes and mechanisms shaping children’s socioemotional development. Investigating the role of paternal mental health and sensitivity, as well as the influence of broader family sociodemographic and environmental factors, would further enrich this area of research. In addition, given the high comorbidity between maternal depression and anxiety, examining how co-occurring anxiety may uniquely or interactively affect maternal sensitivity and infant ER would be an important direction for future work.

The finding that infants of depressed mothers showed lower object-attend in the Fear task but not in the SFP might reflect differences in the lengths of the two tasks and structure. That is, the longer still-face episode may eventually prompt object-looking simply due to lack of stimulation. Future work should examine ER dynamically over the full 120 s of the still-face episode to better capture these shifts.

Intervention studies specifically designed to enhance maternal sensitivity in clinically depressed mothers—and to assess subsequent changes in infant ER across multiple emotional contexts—would be invaluable for translating these findings into clinical practice. Clarifying the mechanisms through which maternal sensitivity fosters regulatory skill development is a critical next step. Clinically, our results underscore the importance of interventions that strengthen sensitive caregiving, even in the presence of maternal depression, as these efforts may buffer infants from the negative effects of maternal mood disturbances. Early detection of maternal depression combined with targeted parenting support could help mitigate long-term risks for infants. Moreover, the finding that infants of depressed mothers demonstrated specific deficits in attentional regulation (e.g., monitoring, gaze aversion, object-attend) suggests that interventions might be especially effective if they equip mothers with strategies to scaffold these specific regulatory skills, thereby supporting infants’ broader socioemotional development.

## 6. Conclusions

Our study confirms that maternal depression poses a significant risk for the development of infant emotion regulation but offers an important nuance: this risk is profoundly shaped by the quality of maternal sensitivity and contextual arousal. By demonstrating a moderating role for maternal sensitivity on infant ER, this research provides strong support for the development of dyadic interventions that focus on enhancing maternal sensitivity to improve ER outcomes in infants of depressed mothers. These findings contribute to our theoretical understanding of early regulatory development, and they hold significant promise for clinical application. The differential patterns of ER behaviors across the SFP and Fear task further highlight the context-dependent nature of infant regulatory strategies, reinforcing the need for multi-contextual assessments.

## Figures and Tables

**Table 1:** Sociodemographic characteristics of the matched sample (N = 120).

		Depressed	Nondepressed	F/χ^2^
		*n* = 60	*n* = 60	
Mother				
	Age at infant’s birth (years)	31.72 (4.97)	31.42 (4.21)	*F*(1,119) = 0.12, *ns*
	Education	5.92 (0.90)	6.15 (0.76)	*F*(1,119) = 2.34, *ns*
	Employment status (% employed)	35.0	41.7	χ^2^(1) = 0.56, *ns*
	Hours employed per week outside home	13.25 (17.28)	12.15 (16.55)	*F*(1,118) = 0.12, *ns*
	Ethnicity (% Non-Hispanic White)	68.3	70.0	χ^2^(1) = 1.74, *ns*
	Hollingshead SES	53.63 (9.18)	56.31 (8.42)	*F*(1,115) = 2.67, *ns*
	Lives with child’s biological father (%)	95.0	95.0	χ^2^(1) = 0.01, *ns*
Infant				
	Age (days)	154.87 (7.40)	153.57 (6.77)	*F*(1,119) = 1.01, *ns*
	Birthweight (g)	3250.52 (443.57)	3284.20 (517.38)	*F*(1,118) = 0.15, *ns*
	Gender (% female)	35.0	38.3	χ^2^(1) = 0.14, *ns*
	Birth order (% firstborn)	68.3	50.0	χ^2^(1) = 4.17, *p* = 0.041

*ns*: non-significant.

**Table 2:** Results of UNIANOVA to examine the effects of maternal depression on infant ER across the two contexts.

			Nondepressed			Depressed					
		Mean (*M*)	95% CI [Lower, Upper]	Std. Error (*SE*)	Mean (*M*)	95% CI [Lower, Upper]	Std. Error (*SE*)	*F*(1,118)	*B* *	*p*	η^2^
SFP											
	Protest	−1.59	[−1.88, −1.31]	0.14	−1.51	[−1.79, −1.22]	0.14	0.19	−0.09	0.662	0.00
	Monitor	−0.67	[−0.82, −0.52]	0.08	−0.96	[−1.11, −0.81]	0.08	7.01	0.29	0.009	0.06
	Gaze Aversion	−0.68	[−0.81, −0.55]	0.07	−0.95	[−1.08, −0.82]	0.07	8.54	0.27	0.004	0.07
	Object Attend	−0.43	[−0.64, −0.22]	0.11	−0.40	[−0.61, −0.19]	0.11	0.05	−0.03	0.828	0.00
	Self-Soothing	−0.28	[−0.47, −0.10]	0.09	−0.27	[−0.45, −0.08]	0.09	0.01	−0.01	0.916	0.00
Fear Task											
	Fear	0.69	[0.50, 0.87]	0.09	0.96	[0.78, 1.15]	0.09	4.29	−0.28	0.040	0.04
	Gaze Aversion	0.64	[0.57, 0.70]	0.03	0.52	[0.45, 0.59]	0.03	5.76	0.12	0.018	0.05
	Object Attend	0.88	[0.83, 0.94]	0.03	0.78	[0.73, 0.84]	0.03	6.54	0.10	0.012	0.05
	Self-Soothing	0.28	[0.19, 0.37]	0.05	0.38	[0.29, 0.57]	0.05	2.30	−0.09	0.101	0.01

* Note: The comparison group is Depressed.

**Table 3:** Correlations between maternal sensitivity and infant ER across the two contexts (N = 120).

	Still-Face Paradigm (SFP)						Fear Task			
	Maternal Sensitivity	Protest	Monitor	Gaze Aversion	Object Attend	Self-Soothing	Fear	Gaze Aversion	Object Attend	Self-Soothing
Maternal Sensitivity	1	−0.09	0.18 *	0.28 **	0.12	0.15	−0.14	0.45 **	0.30 **	0.02
Protest		1	−0.23 **	−0.18 *	−0.37 **	−0.22 *	0.09	−0.02	0.03	0.09
Monitor			1	−0.09	−0.22 *	0.03	−0.17	0.24 **	0.13	0.01
Gaze Aversion				1	−0.01	0.01	−0.11	0.05	0.09	0.02
Object Attend					1	0.21 *	−0.15	0.05	0.01	−0.01
Self-Soothing						1	0.01	0.07	0.0	0.01
Fear							1	−0.15	−0.24 **	0.07
Gaze Aversion								1	0.15	−0.01
Object Attend									1	−0.03
Self-Soothing										1

Note: * *p* < 0.05, ** *p* < 0.01.

**Table 4:** Results of regressions to examine the interactive effects of maternal depression X sensitivity on infant ER.

		*B*	95% CI [Lower, Upper]	Std Error (*SE*)	*R*^2^ Change	*p*	Effect Size (*f*^2^)
Still-Face							
	Protest	0.22	(−0.790, 1.233)	0.51	0.01	0.665	0.01
	Monitor	0.13	(−1.156, −0.106)	0.23	0.12	0.572	0.14
	Gaze Aversion	0.63	(−0.325, 0.587)	0.27	0.13	0.019	0.15
	Object Attend	0.23	(−0.520, 0.971)	0.38	0.02	0.551	0.02
	Self-Soothing	0.56	(−0.094, 1.207)	0.33	0.05	0.093	0.05
							
Fear task	Fear	−0.23	(−0.895, 0.434)	0.34	0.05	0.493	0.05
	Gaze Aversion	0.27	(0.052, 0.485)	0.11	0.26	0.016	0.35
	Object Attend	0.25	(0.060, 0.437)	0.10	0.17	0.010	0.20
	Self-Soothing	0.11	(−0.222, 0.434)	0.17	0.00	0.524	0.00

Note: We entered maternal depression X sensitivity in Step2, after entering maternal depression and maternal sensitivity individually in Step 1.

**Table 5:** Mixed model results using GLM on infant ER strategies (z-scores) across SFP and Fear task.

		Gaze Aversion			Object Attend			Self-Soothing		
		*F*	*p*	η^2^	*F*	*p*	η^2^	*F*	*p*	η^2^
Step 1	Context	0.00	1.00	0.00	0.00	0.952	0.00	0.01	1.000	0.00
Step 1	Depression × Context	0.12	0.730	0.00	0.00	0.995	0.00	3.95	0.049	0.04
Step 2	Sensitivity × Context	2.00	0.160	0.02	1.16	0.283	0.01	1.14	0.288	0.01
Step 3	Depression × Sensitivity × Context	1.35	0.248	0.01	0.46	0.498	0.00	1.17	0.196	0.02

## Data Availability

Data will be made available upon reasonable request. Restrictions apply to the datasets. The datasets presented in this article are not readily available because they are the property of NIH.

## Abbreviations

SFP	Still-Face Paradigm
ER	Emotion Regulation
PPD	Postpartum Depression
